# Depressive Disorders and Religious Engagement in Very Old People

**DOI:** 10.1177/2333721419846576

**Published:** 2019-05-10

**Authors:** Sofia Strinnholm, Yngve Gustafson, Johan Niklasson

**Affiliations:** 1 Umeå University, Sweden

**Keywords:** depression, aged, 80 and above, religion, salutogenesis

## Abstract

**Objective:** To examine associations between religious engagement and depressive disorders in very old people. **Method:** This cross-sectional study uses data from the Umeå 85+/Gerontological Regional Database (GERDA) study. Every other 85-year-old, every 90-year-old, and everyone more than 95 years from eight municipalities in northern Sweden and Finland were invited: 1,014 persons accepted participation. Data were gathered using questionnaires and assessment scales during structured home visits. **Results:** The prevalence of depressive disorders was 35.8%. In a logistic regression model, several factors were adjusted for, such as demographic variables including social factors, diseases, and cognitive and physical functional level. A high level of self-reported religious engagement was independently associated with not having depressive disorders (odds ratio [OR] = 0.58, confidence interval [CI] = [0.38, 0.89]). After stratifying by gender, religious engagement was only significant for women (OR = 0.49, CI = [0.29, 0.82]). **Discussion:** There is an association between a high level of religious engagement and being free from diagnosis of depressive disorders among very old women.

## Introduction

The world’s population is growing increasingly older. The age group that is growing fastest are those aged 60 years and above, comprising 13% of global population and estimated to double by the year 2050. The age group of 80 years and above comprises 2% of population and is estimated to triple by 2050 (United Nations Population Division, 2017). In Sweden, half of those who died in 2015 had celebrated their 83rd birthday (Statistics Sweden, 2016); however, even though the very old are increasing in numbers and ratio of the population, they are seldom included in research.

Depression is important from a global health perspective, particularly in middle and high income countries where it is the number one cause of burden of disease (World Health Organization, 2008). In very old people, depression is often underdiagnosed (Collerton, Davies, & Jagger, 2010; Stek, Gussekloo, Beekman, van Tilburg, & Westendorp, 2004) and undermedicated (Bergdahl et al., 2005; Stek et al., 2004), and many in this age group are still depressed despite treatment (Bergdahl et al., 2005). Very old people often have reduced functional or cognitive capacities and are living alone, which is associated with a higher prevalence of depression (Sjoberg et al., 2017). Luppa et al. (2012) found in a systematic review a prevalence of depressive disorders from 20% to 25% among those older than 85 years and 30% to 50% among those 90 years and above.

One variable that may protect against depression in later life is religiosity. Law and Sbarra (2009) found that going to church had a protective effect against depression among old people (mean age = 75.6 years). Braam et al. (2004) found that church attendance was negatively associated with the trajectory of depressive symptoms, even after adjusting for confounders (mean age = 68.1 years). Koenig (2009) found that religion can act as a coping resource during psychological and social stress. You et al. (2009) report that older Koreans who have a higher self-rated religiosity and spirituality have a lower level of depression and a higher level of general health, even after correcting for demographic variables and living conditions. Yoon and Lee (2006) saw that spirituality and religiosity were negatively associated with depression, but positively associated with life satisfaction. Even if religious convictions and religious practice can be sources of comfort, hope, and meaning, they are often mixed with neurotic and psychotic disorders, which complicate the assessment of whether it is a resource or a burden (Koenig, 2009).

Another research focus has been on whether the effect religion has on depression applies to both women and men. Garfield, Isacco, and Sahker (2013) saw that there are fewer men who are religious, but McFarland (2010) found that men have bigger psychological health-related advantages to religious beliefs than women do. Vaillant found a higher religious involvement in older men with major depression or who had experienced several negative life events (Vaillant, Templeton, Ardelt, & Meyer, 2008). In a community composed primarily of members of the Church of Jesus Christ of Latter-Day Saints (mean age = 75 years), a higher religious involvement was associated with major depression in men, whereas it reduced the risk of depression in women (Norton et al., 2006).

Engagement in voluntary organizations together with interpersonal trust can be used to assess individual-level social capital according to Nyqvist. She found that in persons aged 65 to 80 years, self-rated health was significantly associated with individual-level social capital (Nyqvist, Nygård, & Steenbeek, 2013). The social capital gained from involvement in a religious organization may therefore partly explain the salutogenic effect of a high level of religious engagement.

A challenge for research on religious beliefs and practices is the wide variety of measures being used. Religious involvement often comprises many aspects, but often church attendance is included (Braam et al., 2004; Law & Sbarra, 2009; McFarland, 2010). However, very old people may have problems attending church services, but they are often still engaged in their religious beliefs and often continue to practice on their own with prayer, daily devotion, and sacred reading (You et al., 2009). In Sweden, 88% of those above the age of 85 years are members of the Swedish Church; however, there are no available statistics on their level of church attendance (Public relations officer at The Swedish Church, personal communication, January 17, 2018).

The above studies have focused primarily on people who are between 70 and 80 years old, and we were unable to find any studies that explore the connection between religious engagement or involvement and depression in people above the age of 85 years.

Our aim was to test whether there is an association between religious engagement and depression in very old people.

## Method

This study has focused on depressive disorders and factors that may prevent it in very old people and uses data from the Umeå 85+/Gerontological Regional Database (GERDA) and resource center. GERDA is a project that since the year 2000 has gathered data every 5 years on factors that can help achieve and maintain a good aging. For this study we used the Umeå 85+/GERDA data collections from 2005 and 2010. Neither the data from 2000 nor 2015 were used because questions on existential topics were not added until 2005, and the results from 2015 had not yet been validated.

Some participants were interviewed in both 2005 and 2010. To avoid duplicate answers, we identified participants with missing answers from 2005 to the questions about religious engagement and/or depressive disorders. If there were registered answers in 2010, we included those instead of the ones from 2005. If a participant had answered the questionnaire in both 2005 and 2010, we chose to use their answer from 2005.

### Participants

**Figure 1.**
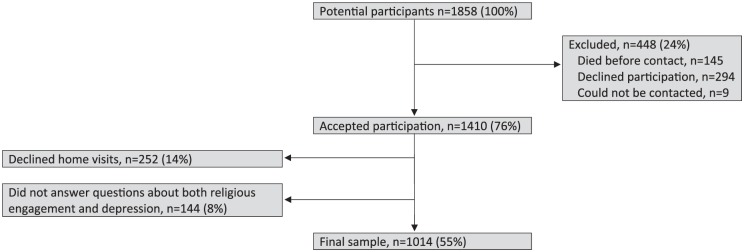
Selection of final sample.

We had 1,858 potential participants, 145 died before contact, 294 declined participation, and 9 could not be contacted. Of the 1,410 who accepted participation, 252 declined home visits and 144 did not answer the questions about religious engagement and depressive disorders. The final sample consisted of 1,014 participants (Figure 1).

Those who died before contact, declined participation, or could not be contacted (*n* = 448) were more likely to be women (74.8% compared with 68.7%, *p* = .01), but there was no statistically significant difference in age when compared with those who accepted participation. Individuals who accepted participation but who were not included in the final sample (*n* = 396) were more likely to be older (mean age = 90.30 years, *SD* = 4.804, *p* = .015), but there was no statistically significant difference in gender, number of drugs, or whether or not they lived alone, when compared with the final sample.

### Procedure

GERDA’s participants were from municipalities (Umeå, Dorotea, Malå, Sorsele, Storuman, and Vilhelmina) in Västerbotten County in Northern Sweden or municipalities (Vasa, Korsholm, Malax, Korsnäs) in the Ostrobothnia region in Western Finland. Every other 85-year-old, every 90-year-old, and everyone above the age of 95 years from the Swedish National Tax Board and the Finnish Population Register were invited to join. To determine which of the 85-year-olds would be invited, a lot was drawn to decide between even or odd numbers in the population registers. The oldest participants were interviewed first.

The selected participants were sent a letter with information about the study, and then a research assistant called them to inform them of the study and asked whether or not they wanted to participate. If they agreed to participate, a home visit was booked. In cases of reduced cognitive function, their next of kin was asked for informed consent. In cases where the participant was in institutional care, the personnel were contacted first to determine the cognitive ability, thereafter the participant themselves or their next of kin were contacted.

The structured interview and the health check were done in one, sometimes two, home visits. The interview took approximately 2 hr to complete and was conducted in the participant’s native language, either Swedish or Finnish. If the participant was in institutional care, the personnel were also interviewed, after consent from the participant. The interview followed a predefined protocol that included general questions about demographic, medical, social, societal, and existential aspects of their lives, as well as medical history and assessment scales. Medical records were later used to validate data.

Those who conducted the interviews were a physician, nurse, physiotherapist, or medical student and were specially trained in interviewing old people. The first interview every interviewer conducted was supervised by an experienced colleague to ensure the quality.

### Measures

Geriatric Depression Scale-15 (GDS-15) was used to screen for depressive symptoms. Scores of five or more indicate a need for further assessment. GDS-15 consists of 15 questions on mood, lack of energy, anxiety, and withdrawnness and was developed for use in geriatric persons (Conradsson et al., 2013; Craen, Heeren, & Gussekloo, 2003; Dias et al., 2017; Marc, Raue, & Bruce, 2008). The Philadelphia Geriatric Center Morale Scale (Lawton, 1975) and the Organic Brain Syndrome Scale (Björkelund, Larsson, Gustafson, & Andersson, 2006) were also used to assess depressive and other psychiatric symptoms. If the interviewer was either a physician or a specifically trained medical student, the Montgomery-Åsberg Depression Rating Scale (MADRS) was used as well (Montgomery & Åsberg, 1979).

The diagnosis of depressive disorder was validated by an experienced geriatrician and based on depressive symptoms and medical treatment for depression. Depressive disorders were diagnosed according to the criteria used in the *Diagnostic and Statistical Manual of Mental Disorders* (4th ed.; American Psychiatric Association, 1994). Participants who had ongoing treatment for depression were considered to have depressive disorders regardless of their scores on the assessment scales. Aside from major depression, the following were also considered depressive disorders: minor depression, dysthymia, depression due to side effects of medication, and depression due to general medical conditions.

Religious engagement was measured by asking participants “Are you a member/active in any religious or spiritual organization/congregation?” with the following options: not a member, passive member, active member, very active member. We dichotomized these into two categories: low engagement (not a member/passive member) and high engagement (active/very active member).

Mini Mental State Examination (MMSE) is an assessment scale used to assess cognitive function (memory, learning, orientation). MMSE has a maximum of 30 points, and scores below 24 indicate an impaired cognition (Folstein, Folstein, & McHugh, 1975).

Barthel’s Index of Activities of Daily Living (ADL) assesses personal ADL in eight different activities. Scores are summed up from 0 to 20 where 20 indicates complete independence in personal ADL (Mahoney & Barthel, 1965). Barthel’s Index of ADL has been shown to have good validity and reliability (McDowell, 2006).

Analgesic was defined as regular use of opioids, paracetamol, and/or nonsteroidal anti-inflammatory drugs (NSAIDs). Visual impairment was defined as not being able to read a 5 mm text from a distance of 30 cm, with or without glasses. Hearing impairment was defined as not being able to hear someone speaking at normal level from a distance of 1 m, with or without hearing aids.

The characteristics chosen as confounders were either known to or could through logical reasoning be hypothesized to be associated with depression in very old people.

### Statistics

IBM SPSS Statistics version 24 was used for all the statistical analyses. The bivariate tests used were χ^2^ test, Student’s *t* test, Fisher’s test, and correlation. The *p* values <.05 were considered statistically significant.

All confounding variables with *p* value of ≤.1 and Spearman’s rho <0.50 were added to a logistic regression model. Barthel’s ADL index had a high multicollinearity with “times outdoors in the past week” (Spearman’s ρ = 0.617), and the latter was therefore not included in the regression model. Both Barthel’s ADL index and MMSE had a high multicollinearity (Spearman’s ρ = 0.556), and we chose to retain them both anyway because we believed that they affect the risk of depressive disorders in different ways. Although we found a high multicollinearity among some of the social factors (living in institutional care and feelings of loneliness) using χ^2^ tests, none of them were excluded from the regression model for the same reason. This means that the effects of the social factors cannot be distinguished from each other.

### Ethics

In the Ostrobothnia region, the Ethical Committee at Vaasa Central Hospital in Finland approved the implementation of the home visits (05-87 and 10-54). In Västerbotten, the Regional Ethical Review Board in Umeå, Sweden, approved the implementation of the home visits (99-326, 05-063M, 09-178M, 2013-17-31M, and 14-221-31M). An additional application for processing the data and writing the report has been approved by the Regional Ethical Review Board in Umeå, Sweden (2017/415-31).

## Results

### Univariate Analyses

The prevalence of depressive disorders was 35.8%, an indication that in the very old people, depressive disorders are common. Depressive disorders were more common in women than men (38.7% compared with 29.9%).

### Bivariate Analyses

Table 1 shows the characteristics of the participants who had depressive disorders and those who did not. Those who had depressive disorders were significantly older but were most likely to be in the group that was 90 years old. They were also more likely to be Swedish, to be women, to have spent fewer years in school, to live alone, and to live in institutional care. They were more likely to experience feelings of loneliness and to feel unsafe. Participants with depressive disorders were also more likely to have had a stroke, to have heart failure, sleep disorders, visual impairment, a higher number of medications for regular use, and were more likely to regularly use analgesics. They were more likely to be physically disabled (lower Barthel’s ADL score) and to have cognitive impairment (lower MMSE score). Participants with depressive disorders were less likely to exercise regularly and had gone outdoors fewer times in the past week. A high level of religious engagement was more likely in participants free from depressive disorders.

**Table 1.** Basic Characteristics of the Participants With Depressive Disorders and Without Depressive Disorders (*n* = 1,014).

	Depressive disorders (*n* = 363)	Not depressed (*n* = 651)	*p* value
Age (years)	90.1 ± 4.5 (*n* = 363)	89.4 ± 4.7 (*n* = 651)	.030
Age group (years)	(*n* = 363)	(*n* = 651)	.003
85^a^ (*n* = 444)	30.2% (*n* = 134)	69.8% (*n* = 310)	
90^a^ (*n* = 311)	41.5% (*n* = 129)	58.5% (*n* = 182)	
95+^a^ (*n* = 259)	38.6% (*n* = 100)	61.4% (*n* = 159)	
Sex (women)	72.7% (*n* = 363)	64.4% (*n* = 651)	.008
Years in school	6.9 ± 2.1 (*n* = 335)	7.5 ± 2.7 (*n* = 611)	.001
Geographical area	(*n* = 363)	(*n* = 651)	.042
Sweden^a^ (*n* = 644)	38.2% (*n* = 246)	61.8% (*n* = 398)	
Finland^a^ (*n* = 370)	31.6% (*n* = 117)	68.4% (*n* = 253)	
Living alone	86.0% (*n* = 357)	74.2% (*n* = 650)	<.001
Living in institutional care	45.7% (*n* = 363)	21.1% (*n* = 650)	<.001
Feelings of loneliness	63.1% (*n* = 328)	36.1% (*n* = 615)	<.001
Feeling unsafe	8.8% (*n* = 319)	3.2% (*n* = 601)	<.001
Deceased child/children	21.4% (*n* = 337)	19.0% (*n* = 622)	.422
Hypertension	72.5% (*n* = 363)	67.7% (*n* = 651)	.136
Stroke	16.0% (*n* = 363)	9.1% (*n* = 651)	.001
Heart failure	36.9% (*n* = 363)	27.5% (*n* = 651)	.002
Chronic lung disease	18.2% (*n* = 363)	15.1% (*n* = 651)	.227
Malignancy	8.8% (*n* = 363)	6.3% (*n* = 651)	.174
Sleep disorder	54.3% (*n* = 363)	31.3% (*n* = 651)	<.001
Visual impairment	22.0% (*n* = 350)	11.7% (*n* = 639)	<.001
Hearing impairment	18.4% (*n* = 359)	15.5% (*n* = 644)	.281
Regular use of analgesics (excluding ASA)	43.5% (*n* = 363)	26.9% (*n* = 650)	<.001
Number of drugs	8.2 ± 3.9 (*n* = 363)	5.8 ± 3.5 (*n* = 650)	<.001
Body mass index	25.9 ± 4.8 (352)	25.7 ± 4.1 (*n* = 629)	.581
Self-reported regular exercise	62.1% (*n* = 335)	73.1% (*n* = 625)	.001
Times outdoors in the past week	3.1 ± 4.5 (*n* = 340)	5.0 ± 4.5 (*n* = 628)	<.001
Barthel’s ADL index	15.6 ± 5.1 (*n* = 362)	17.9 ± 4.1 (*n* = 649)	<.001
Mini Mental State Examination	19.8 ± 6.8 (*n* = 350)	23.1 ± 5.9 (*n* = 638)	<.001
High religious engagement	17.4% (*n* = 363)	24.0% (*n* = 651)	.018

*Note.* χ^2^ test, Fisher’s test, and Student’s *t* test were used. Age, years in school, times outdoors in the past week, Barthel’s ADL index, and MMSE are presented as mean values ± standard deviation. ASA = acetylsalicylic acid; ADL = activities of daily living; MMSE = Mini Mental State Examination.

a Presented as percentage of line total.

In Table 2, the characteristics of the participants with a high and low level of religious engagement are shown, respectively. The data showed that 21.6% of the participants had a high level of religious engagement. Participants with a high religious engagement were more likely to be women, live alone, exercise regularly, have been outdoors more often, have a higher Barthel’s ADL score and less often diagnosed with depressive disorders.

**Table 2.** Basic Characteristics of the Participants With Low and High Religious Engagement (*n* = 1,014).

	Low religious engagement (*n* = 795)	High religious engagement (*n* = 219)	*p* value
Age (years)	89.6 ± 4.6 (*n* = 795)	89.8 ± 4.68 (*n* = 219)	.612
Age group (years)	(*n* = 795)	(*n* = 219)	.432
85^a^ (*n* = 444)	79.5% (*n* = 353)	20.5% (*n* = 91)	
90^a^ (*n* = 311)	75.9% (*n* = 236)	24.1% (*n* = 75)	
95+^a^ (*n* = 259)	79.5% (*n* = 206)	20.5% (*n* = 53)	
Sex (women)	65.3% (*n* = 795)	74.9% (*n* = 219)	.009
Years in school	7.3 ± 2.6 (*n* = 744)	7.3 ± 2.2 (*n* = 202)	.950
Geographical area	(*n* = 795)	(*n* = 219)	.589
Sweden^a^ (*n* = 644)	77.8% (*n* = 501)	22.2% (*n* = 143)	
Finland^a^ (*n* = 370)	79.5% (*n* = 294)	20.5% (*n* = 76)	
Living alone	76.2% (*n* = 789)	86.2% (*n* = 218)	.002
Living in institutional care	29.6% (*n* = 794)	31.1% (*n* = 219)	.740
Feelings of loneliness	45.3% (*n* = 731)	46.2% (*n* = 212)	.869
Feeling unsafe	5.2% (*n* = 717)	4.9% (*n* = 203)	1.000
Deceased child/children	19.3% (*n* = 748)	21.8% (*n* = 211)	.470
Depressive disorder	37.7% (*n* = 795)	28.8% (*n* = 219)	.018
Hypertension	70.4% (*n* = 795)	65.8% (*n* = 219)	.211
Stroke	10.7% (*n* = 795)	14.6% (*n* = 219)	.137
Heart failure	30.9% (*n* = 795)	30.6% (*n* = 219)	.987
Chronic lung disease	16.5% (*n* = 795)	15.1% (*n* = 219)	.691
Malignancy	7.5% (*n* = 795)	5.9% (*n* = 219)	.503
Sleep disorder	38.6% (*n* = 795)	42.9% (*n* = 219)	.282
Visual impairment	15.9% (*n* = 773)	13.4% (*n* = 216)	.430
Hearing impairment	16.6% (*n* = 785)	16.5% (*n* = 218)	1.000
Regular use of analgesics (excluding ASA)	33.4% (*n* = 794)	31.1% (*n* = 219)	.571
Number of drugs	6.6 ± 3.8 (*n* = 794)	6.6 ± 4.1 (*n* = 219)	.910
Body mass index	25.8 ± 4.2 (*n* = 770)	25.6 ± 4.1 (*n* = 211)	.575
Self-reported regular exercise	66.3% (*n* = 753)	80.2% (*n* = 207)	<.001
Times outdoors in the past week	4.2 ± 4.3 (*n* = 758)	5.2 ± 5.5 (*n* = 210)	.005
Barthel’s ADL index	16.9 ± 4.7 (*n* = 792)	17.6 ± 4.1 (*n* = 219)	.033
Mini Mental State Examination	21.9 ± 6.5 (*n* = 775)	22.4 ± 5.9 (*n* = 213)	.328

*Note.* χ^2^ test, Fisher’s test, and Student’s *t* test were used. Age, years in school, times outdoors in the past week, Barthel’s ADL index and MMSE are presented as mean values ± standard deviation. ASA = acetylsalicylic acid; ADL = activities of daily living; MMSE = Mini Mental State Examination.

a Presented as percentage of line total.

### Multivariate Analyses

In Table 3, a multiple logistic regression with depressive disorders as the outcome is shown. A high religious engagement was found to be independently negatively associated with depressive disorders after adjusting for several demographic variables such as social factors, diseases, cognitive, and physical functional level using a logistic regression model (odds ratio [OR] = 0.6, *p* = .03). Factors that were significantly positively associated with depressive disorders were sleep disorders, a higher number of medications for regular use, decreased physical function, and decreased cognitive function. Feelings of loneliness were also significantly positively associated with depressive disorders, but because of a high multicollinearity with other social factors, we cannot determine how much of an effect the factor has independently.

**Table 3.** Multiple Logistic Regression With Depressive Disorders as the Outcome (*n* = 812).

	Multiple logistic regression		
	(*n* = 812)		
	Model fit: H&L = 0.289. Nagelkerke = 0.309		
	OR	95% CI	*p* value
Age	0.959	[0.917, 1.003]	.066
Sex (women)	1.121	[0.757, 1.660]	.570
Years in school	0.952	[0.883, 1.026]	.197
Geographical area	1.142	[0.787, 1.658]	.483
Living alone	0.947	[0.575, 1.559]	.830
Living in institutional care	0.744	[0.462, 1.198]	.223
Feelings of loneliness	3.054	[2.122, 4.396]	<.001
Feeling unsafe	1.532	[0.758, 3.098]	.235
Stroke	1.395	[0.818, 2.379]	.221
Heart failure	0.864	[0.576, 1.296]	.480
Sleep disorder	2.259	[1.585, 3.220]	<.001
Visual impairment	1.173	[0.703, 1.959]	.541
Regular use of analgesics (excluding ASA)	0.924	[0.617, 1.383]	.700
Number of drugs	1.124	[1.064, 1.186]	<.001
Self-reported regular exercise	0.999	[0.686, 1.456]	.996
Barthel’s ADL index	0.931	[0.876, 0.990]	.022
Mini Mental State Examination	0.934	[0.894, 0.976]	.002
Religious engagement	0.581	[0.379, 0.891]	.013

*Note.* ASA = acetylsalicylic acid; ADL = activities of daily living.

The majority of the sample (67%) was women, as were participants with a high religious engagement, and participants with depressive disorders. To further explore any gender differences, we did a multiple logistic regression stratified into men and women (see Table 4). We found that in women with depressive disorders, there was a significant association with fewer years in school, feelings of loneliness, sleep disorders, a higher number of medications for regular use, lower MMSE score, and a low religious engagement. For men, only feelings of loneliness and a lower ADL score were significantly associated with depressive disorders. The data, therefore, only support a negative association between high religious engagement and depressive disorders in women, not in men. After stratifying the sample by gender, religious engagement was only significant for women (OR = 0.49, confidence interval [CI] = [0.29, 0.82]).

**Table 4.** Multiple Logistic Regression With Depressive Disorders as the Outcome Stratified by Gender (*n* = 812).

	Women			Men		
	(*n* = 535)			(*n* = 277)		
	Model fit: H&L = 0.433. Nagelkerke = 0.344			Model fit: H&L = 0.226. Nagelkerke = 0.277		
	OR	95% CI	*p* value	OR	95% CI	*p* value
Age	0.952	[0.902, 1.005]	.076	0.964	[0.881, 1.054]	.421
Years in school	0.891	[0.810, 0.981]	.019	1.053	[0.941, 1.179]	.367
Geographical area	1.355	[0.866, 2.122]	.184	0.766	[0.364, 1.612]	.483
Living alone	0.688	[0.324, 1.463]	.331	1.350	[0.643, 2.834]	.428
Living in institutional care	0.801	[0.446, 1.441]	.459	0.604	[0.252, 1.449]	.259
Feelings of loneliness	2.959	[1.903, 4.599]	<.001	3.311	[1.643, 6.673]	.001
Feeling unsafe	1.729	[0.735, 4.068]	.210	1.634	[0.439, 6.078]	.464
Stroke	1.323	[0.682, 2.564]	.407	1.432	[0.537, 3.821]	.473
Heart failure	0.906	[0.555, 1.478]	.692	0.702	[0.314, 1.567]	.387
Sleep disorder	2.901	[1.867, 4.507]	<.001	1.611	[0.830, 3.126]	.159
Visual impairment	1.039	[0.559, 1.932]	.903	1.301	[0.483, 3.504]	.602
Regular use of analgesics (excluding ASA)	0.722	[0.444, 1.172]	.187	1.388	[0.615, 3.131]	.430
Number of drugs	1.158	[1.084, 1.238]	<.001	1.062	[0.958, 1.179]	.253
Self-reported regular exercise	0.897	[0.568, 1.417]	.641	1.255	[0.607, 2.598]	.540
Barthel’s ADL index	0.937	[0.871, 1.009]	.084	0.874	[0.768, 0.995]	.042
Mini Mental State Examination	0.932	[0.882, 0.985]	.012	0.938	[0.864, 1.018]	.124
Religious engagement	0.492	[0.294, 0.823]	.007	0.794	[0.340, 1.857]	.595

*Note.* ASA = acetylsalicylic acid; ADL = activities of daily living.

A relatively high percentage (20%) of the sample had missing variables in the logistic regression and was therefore not included. To explore whether this was systematic, we compared the missing cases with the total sample used in the regression. In general, the participants with missing variables were more likely to be older, have more diseases, more cognitive and physical disability, and were more likely to suffer a depressive disorder (data not shown).

## Discussion

A high level of religious engagement was associated with being free from depressive disorders among very old people. However, when the sample was stratified by gender, religious engagement was only significant for women.

The prevalence of depressive disorders was high, which may partially be explained by the high age of the participants and the inclusion of other depressive disorders than major depression. Other factors that may have contributed to the high prevalence were the high participation rate and the inclusion of those living in institutional care which may have led to a higher burden of comorbidities. We found that depressive disorders were more common in women than men (38.7% compared with 29.9%). In this study, reasons for the gender discrepancy were not examined, but our data suggest a higher burden of disease (more drugs used, more sleep disorders, and lower MMSE scores). Another contributing factor may be that women in Sweden tend to outlive their husbands. In our sample, 89.4% of the women lived alone compared with 55.6% of the men. Therefore, one of the possible reasons for the gender discrepancy found in the current study may be that women are more likely to experience the loss of a partner. Social aspects seem important because loneliness was the only factor related to depressive disorders for both men and women in our multiple logistic regression model stratified by gender.

Other studies with slightly younger participants have also reported that high level of religious engagement is associated with being free from diagnose of depressive disorder (Yoon & Lee, 2006; You et al., 2009). Some studies have found church attendance in particular to be the most important protective factor in religious engagement (Braam et al., 2004; Law & Sbarra, 2009; Sun et al., 2012). However, men in our study did not have an association between religious engagement and being free from a depressive disorder. Previous studies in slightly younger populations (mean age = 74-75 years) have found contradicting results; McFarland (2010) found religious beliefs to give more of a psychological advantage to men than women, whereas Norton found a high religious engagement to be associated with an increased prevalence of depression in men and a reduced prevalence in women (Norton et al., 2006).

Individual-level social capital is associated with self-rated health according to Nyqvist. Engagement in voluntary organizations was one of the two ways in which she assessed social capital on an individual level (Nyqvist et al., 2013). Therefore, the social capital gained from a high religious engagement may be what causes the salutogenic effect found in this study. Contradicting this is that after several social factors were adjusted for in our study, the association between a high level of religious engagement and being free from depressive disorders remained. This suggests that a high level of religious engagement may have a salutogenic effect independently of the social support that a high level of engagement may bring. Another reason for the salutogenic effect may be that being part of an organization might bring a sense of purpose and belonging to the lives of very old people. That sense of purpose and belonging may be especially important because many of them lose their spouses and their friends as well as experience a decline in physical ability. The act of engaging in an activity of their preference may also in itself boost their psychological resilience.

### Strengths and Limitations

Strengths of the study are that there was a high participation rate, the population registers used to invite participants were considered reliable, and the study included both those who reside in community dwellings and those who live in institutional care. The participants were representative of those aged 85 years, 90 years, and ≥95 years; however, age was used as a continuous variable despite the participants being stratified in three age groups.

Another strength is that many variables were included in the regression analyses and no exclusion was made based on low cognitive function. The data gathering was extensive and conducted in a structured way in the participants’ own homes by persons who were trained in doing so. An experienced geriatrician assessed the diagnosis of depressive disorder.

A limitation of the study was that it was cross-sectional and therefore unable to determine cause and effect. We were unable to compare religious engagement to engagement in secular organizations, and further studies are needed to see if they have similar salutogenic effects. Participants rated their religious engagement themselves, so the level of engagement that one participant may define as low another participant may define as high. Furthermore, we did not test for differences in personality. The participants’ propensity for participating in the study might correlate with the level of religious engagement; hence, there might be self-selection bias.

The high number of cases with missing data (20%) in the logistic regression model is another limitation. The individuals who were not included in the regression due to missing data had more diseases, more cognitive and physical disabilities, and were more likely to suffer from a depressive disorder. It is therefore plausible that the results may not be applicable to the participants with the highest burden of disease, particularly not those with dementia because the MMSE score was markedly lower in the group that was not included (15.9 points compared with 22.0).

The sample is likely to be representative of people more than the age of 85 years in Västerbotten in Sweden and the Ostrobothnia region in Finland. Two countries being included increases the chances of the results being applicable to other countries, particularly other Nordic countries.

## Conclusion

There is an association between a high level of religious engagement and being free from diagnose of depressive disorders among very old women.
